# Fiber Optic Fabry-Perot Current Sensor Integrated with Magnetic Fluid Using a Fiber Bragg Grating Demodulation

**DOI:** 10.3390/s150716632

**Published:** 2015-07-09

**Authors:** Ji Xia, Qi Wang, Xu Liu, Hong Luo

**Affiliations:** 1 Academy of Ocean Science and Engineering, National University of Defense Technology, Changsha 410073, China; E-Mails: blovexiaji@163.com (J.X.); luohongrong2000@163.com (H.L.); 2 College of Information Science and Engineering, Northeastern University, Shenyang 110819, China; E-Mail: neuliuxu@gmail.com; 3 State Key Laboratory of Synthetical Automation for Process Industries, Northeastern University, Shenyang 110819, China

**Keywords:** fiber Bragg grating, capillary encapsulating, fiber optic Fabry-Perot sensor, fiber current intensity sensor, light intensity demodulation

## Abstract

An optical fiber current sensor based on Fabry-Perot interferometer using a fiber Bragg grating demodulation is proposed. Magnetic fluid is used as a sensitive medium in fiber optical Fabry-Perot (F-P) cavity for the optical characteristic of magnetic-controlled refractive index. A Fiber Bragg grating (FBG) is connected after the F-P interferometer which is used to reflect the optical power at the Bragg wavelength of the interference transmission spectrum. The corresponding reflective power of the FBG will change with different external current intensity, due to the shift on the interference spectrum of the F-P interferometer. The sensing probe has the advantages of convenient measurement for its demodulation, low cost and high current measurement accuracy on account of its sensing structure. Experimental results show that an optimal sensitivity of 0.8522 nw/A and measurement resolution of 0.001 A is obtained with a FBG at 1550 nm with 99% reflectivity.

## 1. Introduction

Current intensity has been considered as one of the most significant parameters in electrical, chemical and physical fields. Fiber optical current sensors have attracted much attention in recent years on account of their many merits such as safety, and low weight when compared with traditional current sensors [1,2].

Since current cannot be directly measured just by an optical fiber, one kind of common measuring method is to measure the magnetic field that the current generates indirectly, for example, through Faraday effects. MF is a kind of colloidal liquid that contains magnetic nanoparticles dispersing in a suitable liquid carrier. MF does not retain magnetization in the absence of an external field. However, it will become highly magnetized in the presence of a magnetic field. As a magneto-optical nano-material, MF has attracted much research interest owing to its distinguished magneto-optical properties such as tunable refractive index [3], Faraday Effect [4], field dependent transmittance [5] and birefringence [6]. Various optical fiber sensors based on MF have been proposed such as optical fiber Sagnac interferometers [7], cladding-etched fiber Bragg grating [8] and fiber taper structures [9]. Therefore, optical current fiber sensors based on interferometry, especially, Fabry-Perot structure interferometer, have been widely developed [10,11]. In 2010, a fiber optic F-P current sensor based on magnetic fluid (MF) was proposed [12]. MF is used as a sensitive medium in fiber optical F-P cavity. However, many of the aforementioned methods like etching and tapering have limitations of fabrication. They could also be classified as one sort in that the measured current would lead to a force or an effect acting on the sensing head. The sensing information was usually obtained by monitoring the wavelength shift of the interference transmission spectrum, in which an expensive optical spectrum analyzer was necessary. At the same time, temperature cross effect on MF refractive index cannot be eliminated, which would influence the measurement accuracy of measurement system.

In this paper, an optical Fabry-Perot current sensor based on the tunable refractive index property of MF was proposed. At the same time, a fiber Bragg grating (FBG) was used as a demodulation element in optical sensing system by measuring the change of the reflective optical power at the Bragg wavelength. When the external current changes, the interference spectrum shifts, which means that the transmission intensity at a fixed wavelength is sensitive to the change of external current. Thus, the measurement can be realized through a light intensity demodulation for a convenient and cheap solution.

## 2. Experimental Section

### 2.1. Experimental Setup

**Figure 1:**
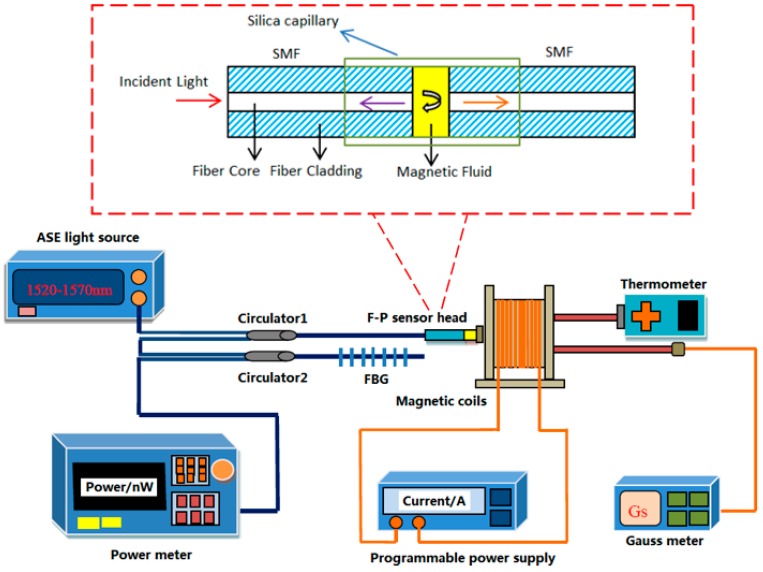
Experimental setup of current sensing system based on the Fabry-Perot (F-P) sensor using a Fiber Bragg grating (FBG) demodulation and the detail structure of F-P sensor.

The experimental setup of the current sensing system based F-P cavity filled with MF using a FBG demodulation is shown in Figure 1, which consists of an amplified spontaneous emission (Golight, OS321886), the F-P sensing head, two circulators, FBG and a light power meter, a programmable power supply (ITECH, IT6154) and a Gauss meter. The detailed structure of the proposed sensor head is shown in the inset of Figure 1.

The amplified spontaneous emission (ASE) has an emission wavelength range covering all the C-band. The magnetic field is generated by the electromagnetic coils, which can be adjusted by using a programmable power supply. The radius of electromagnetic coils is 55 mm, and the number of turns is 750, which can cover well the sensor head aligned in the center and paralleled to the emission surface of the magnet. The F-P sensing head is composed of the MF and two single mode fibers encapsulated into a capillary. FBG is used as a demodulation element in the optical sensing system by measuring the change of the reflective optical power at the Bragg wavelength.

When the external measured current changes, the F-P interference spectrum would shift according to the refractive index of MF changes. This means that the transmission intensity at a fixed wavelength is sensitive to the external current. Therefore, FBG is used to reflect the optical power of the F-P interference spectrum at the Bragg wavelength for current sensing.

### 2.2. Measurement Principle

The principle of the sensing probe is based on the theory of F-P interference and FBG optical properties. In this paper, the sensor is designed as a reflective structure. Because the reflectivity of F-P cavity interfaces is low, the model of multiple beam interference could be simplified by that of the double beam interference. The model can be described as follows:

(1) $$I^{r}=I_{1}+I_{2}+2\sqrt{I_{1}I_{2}}\cos\delta$$

where $I^{r}$ is the reflective interference intensity, $I_{1}$ and $I_{2}$ are the first and second reflection light intensity, respectively. *δ* is the phase difference of the light in the cavity, which could by given by Equation (2):

(2) $$\delta =\frac{4\pi nL}{\lambda }$$

where n is the refractive index of magnetic fluid in the cavity, *L* is the cavity length, and λ is the light wavelength. From Equation (2), we can find two ways to tune the interference spectrum of F-P cavity. One is to change the cavity length and the other is to change the refractive index. In the experiment, we adopted the latter to fill magnetic fluid into the F-P cavity to tune the transmission spectrum. When the measured current changes, the refractive index of MF would change accordingly for the magnet-tunable refractive index property. The relationship between the refractive index of the magnetic fluid and the applied magnetic field under the temperature *T* was investigated by Chen *et al.* [13] which could be described by Equation (3):

(3) $$n_{MF}=\left(n_{s}-n_{0}\right)\left[\cot h\left(\alpha \frac{H-H_{c,n}}{T}\right)-\frac{T}{\alpha \left(H-H_{c,n}\right)}\right]+n_{0},\;\;H>H_{c,n}$$

where, $H_{c,n}$ is the critical value of the magnetic field, $n_{0}$ is the refractive index of the magnetic fluid under the critical magnetic field, $n_{s}$ is the saturation value of the refractive index of magnetic fluid, and *α* is a fitting coefficient. It is noted that, for a given magnetic fluid, $n_{s}$, $n_{0}$, *α*, $H_{c,n}$ are all constants. The refractive index of MF $n_{MF}$ does not change until the magnetic field intensity *H* exceeds a critical field strength $H_{c,n}$. So at a certain temperature *T*, there will be a certain *n versus* a certain *H*. This means a certain *n versus* a certain current. Thus, the current would induce the shift of the F-P spectrum.

When the FBG of the Bragg wavelength $\lambda_{B}$ with reflectivity *R* is connected to the system after the F-P interference, the optical power at the Bragg wavelength of the F-P interference spectrum is reflected. The reflective intensity can be expressed as:

(4) $$I=RI_{B}=R\;\left(\left(I_{1}+I_{2}+2\sqrt{I_{1}I_{2}}\cos\frac{4\pi nL}{\lambda_{B}}\right)\right)$$

Thus, *I* is the corresponding reflective intensity of the FBG at the Bragg wavelength. The FBG is one of the fiber optic devices whose refractive index of fiber core is periodically modulated [14]. The Bragg wavelength $\lambda_{B}$ is given as follows:

(5) $$\lambda_{B}=2n_{\text{eff}}\cdot \text{Λ}$$

where $n_{\text{eff}}$ is effect refractive index of fiber core, Λ is the modulation periodicity of refractive index. The Bragg wavelength shift induced by temperature could be described as Equation (6):

(6) $$\text{Δ}\lambda_{B}=\lambda_{B}\;\left(\alpha_{\text{th}}+\xi \right)\cdot \text{Δ}T=K_{T}^{'}\cdot \text{Δ}T$$

where $\text{Δ}\lambda_{B}$ is Bragg wavelength change, $\alpha_{\text{th}}=0.55\times 10^{-6}/℃$ is the coefficient of thermal expansion of silica, $\xi =8.0\times 10^{-6}/℃$ is the thermo-optical coefficient for 1550 nm, ΔT is the change of temperature, $K_{T}^{'}$ is the total temperature sensitivity of FBG sensor. So the FBG can also be used to compensate the temperature cross-sensitivity effect in further research and application.

In order to obtain optimal results from the sensing system, simulations of the length of F-P cavity and parameters of FBG have been made. According to the interference theory, the wavelength spacing between the transmission adjacent dips is approximately given by:

(7) $$\text{Δλ}=\frac{\lambda^{2}}{nL}$$

By Equation (7) and actual simulations, we know that the smaller the cavity length, the larger the wavelength spacing. Thus, the range of variation of refractive index would be increased. At the same time, the sensitivity would be debased with the decrement of the cavity length. As the interference signal is periodic, the shift of the interference spectrum cannot exceed half of the bandwidth given that the signal is detected by light power meter. Otherwise, the symmetrical interference signal would arise making the measurement unprocurable.

**Figure 2:**
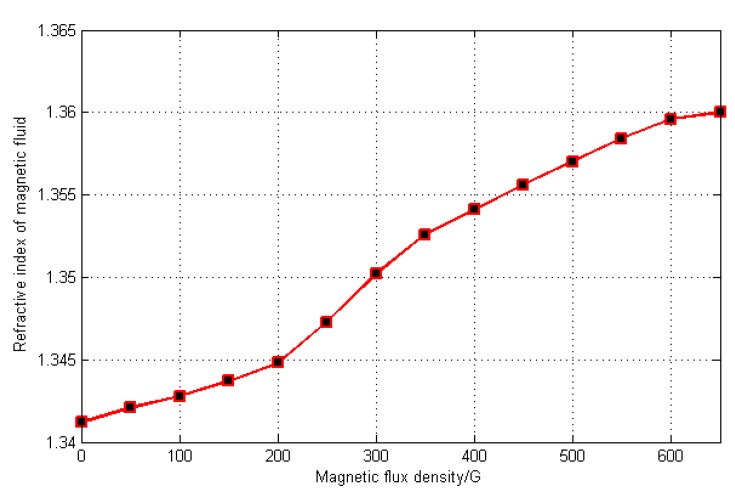
Relationship between the refractive index of magnetic fluid and magnetic flux density.

The variation range of refractive index of MF is 1.3412–1.3600 when the magnetic field is applied to MF [15], which was tested and shown in Figure 2.

**Figure 3:**
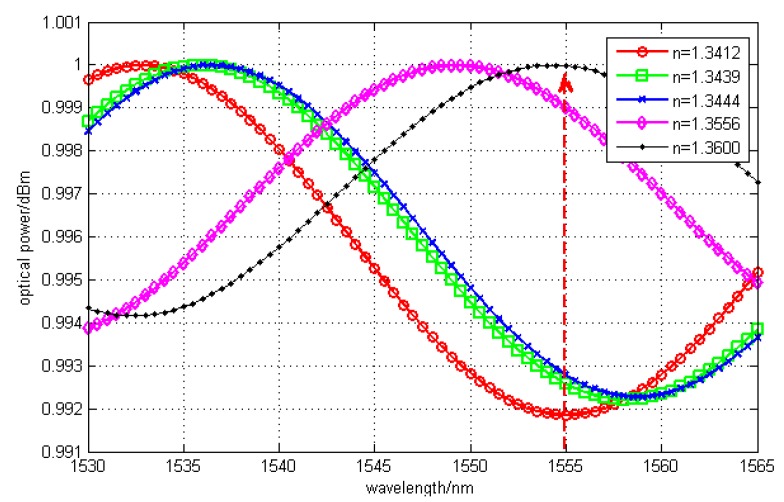
Signals of F-P interference with cavity length of 20 μm.

By simulation, the optimal length of F-P cavity was obtained as 20 μm in the maximum range of refractive index of MF. It is shown in Figure 3.

**Figure 4:**
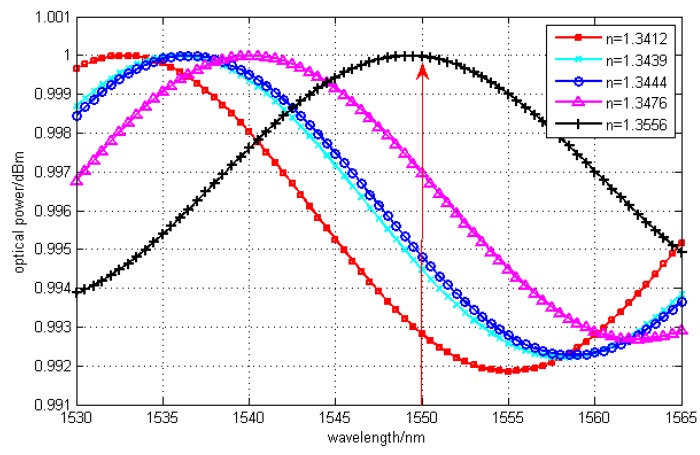
Signals of F-P interference with cavity length of 24μm.

From Figure 3, we can see that all channels of signals could be demodulated when the Bragg wavelength is 1555 nm. Here, the sensitivity of 0.75 nW/A was obtained by fitting the light power at the Bragg wavelength and refractive index. In some practical application, a high sensitivity is demanded and a good degree of linearity is needed. So in the next, a cavity length of 24 μm was set in order to increase the sensitivity and achieve good linearity. However, at the same time, the variation range of refractive index would be decreased inevitably, which means the measurement range would be diminished. Here, the Bragg wavelength was 1550 nm which was easy to fabricate, and the sensitivity was simulated to be enhanced to 1.5 nW/A. The interference spectrum is shown in Figure 4.

Based on above theory, current measurement can be realized by monitoring the reflective power of the FBG at the Bragg wavelength. The change on the F-P interference induced by the change on external current is transformed to the change on the reflective power from the FBG for measurement. Thus, a light power meter could be utilized instead of an expensive OSA for reducing the experimental cost largely.

## 3. Results and Discussion

### 3.1. Fabrication of the Sensor

The sensor proposed in this sensing probe shown in the inset of Figure 1 was fabricated as follows: First, two single mode fibers with a 10 mm part were stripped without coating layer. Then, the prepared fibers were inserted into a glass capillary at both ends slowly, respectively. Second, one fiber was pulled out from one end face slowly and the MF filled the tube by capillary action. Third, the fiber was inserted into the tube again, which would be relatively smooth this time. Finally, both the end faces of the glass capillary were sealed with epoxy glue. All the operations were achieved under a pair of 6-dimension adjustment frames and a microscope.

The water-based MF EMG507 (Ferrotec USA Corporation) was used, and its concentration was 1.8%. Here, magnetic fluid is a kind of colloidal liquid that contains magnetic nanoparticles dispersing in a suitable liquid carrier. MF does not retain magnetization in the absence of an external field. However, it will become highly magnetized in the presence of a magnetic field. The cavity length of F-P sensor was 24 μm, the inner diameter of glass capillary was 125 μm.

### 3.2. Results and Discussion

**Figure 5:**
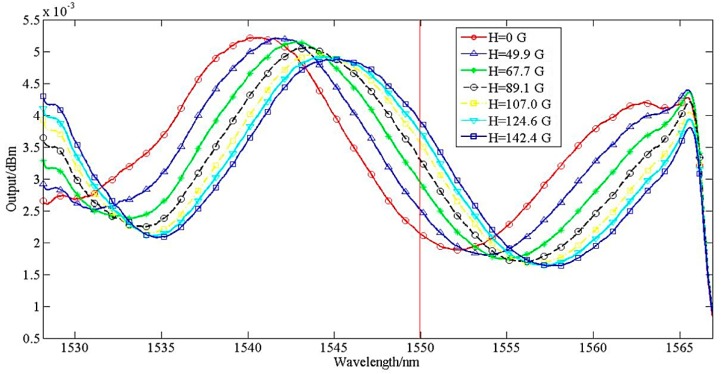
Signals of interference under different current intensity.

In the experiment, the transmission spectrum of the interference has a red-shift with the magnetic field increase. At the Bragg wavelength of FBG, the reflective intensity increased accordingly when the interference spectrum has a red-shift. Figure 5 shows the reflection spectrums of the interference when external current was changed from 0A to 5.5A, which were measured with an OSA in order to see the whole condition of interference.

From Figure 5, we could know that the reflection intensity at the Bragg wavelength of 1550 nm increased when external current increased. It is known that for a given measured current, a fixed magnetic field intensity *H* will be obtained. We also measured the relationship between magnetic field intensity and current via a Gauss meter. After demarcating towards the light power and magnetic field intensity, we could also realize the measurement for magnetic field intensity.

**Figure 6:**
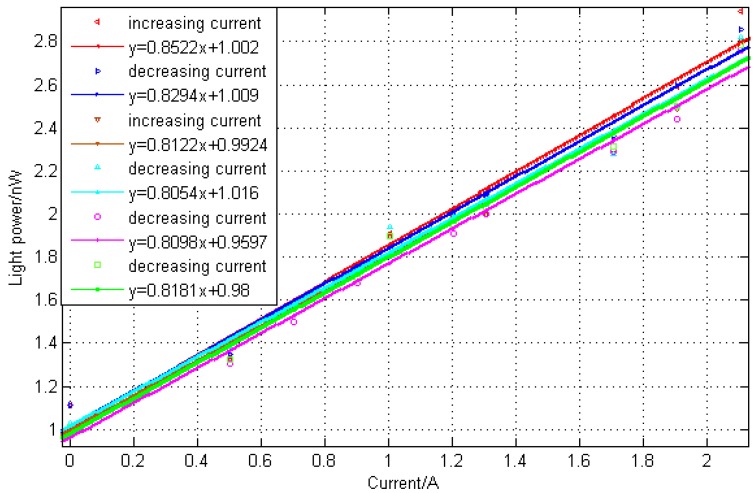
Relationship between light power of the interference with a FBG *versus* current.

Figure 6 showed the reflective power of the interference with FBG at the Bragg wavelength of 1550 nm and the cavity length of 24 μm, which was measured by the power meter. It was discussed by three cycles’ data of increasing and decreasing current. The relationship between the reflective power of the F-P interferometer with a FBG and the measured current can be best fitted as a function such as y=0.8522x+1.002. The fitting degree is as high as $R^{2}=0.9784$. Hence, a sensitivity of 0.8522 nW/A was obtained. Moreover, as the resolution of power meter is 0.001 nW, the resolution of the system towards can be 0.001 A. As for the relationship between magnetic field intensity and current, it can also obtain a sensitivity of 0.0118 nW/G, which is suitable for the measurement of magnetic flux density.

The repeatability of the sensor was affected by the properties of MF. It was investigated by doing three cycles of experiments with increasing and decreasing current. From the fitting results, it can be obtained that the repeatability error of the sensor was 0.0223, 0.0421 and 0.0327, respectively. The hysteresis errors of such measurement were 1.22%, 2.3% and 1.79%, respectively. As a whole, the repeatability was good. In our experiment, the current measurement range is limited as the relationship between the reflective power and current is monotonic. The reflection spectrum of the F-P interferometer is a periodic function of wavelength, so the wavelength shift induced by the different current cannot be more than half of the spectrum width between the two adjacent transmission dips. The largest current measurement range depends on the wavelength spacing between the two wavelength dips and the sensitivity of the F-P interferometer. So in practical applications, a shorter F-P cavity can be utilized to obtain a larger wavelength spacing and increase the sensitivity of interferometer for a better performance.

## 4. Conclusions

In this paper, an optical fiber F-P current sensor integrated with MF using a FBG demodulation was proposed. The F-P interferometer was encapsulated into a capillary using epoxy glue, with which the MF was filled into the tube by capillary action. A FBG was used to demodulate the transmission spectrum. Through monitoring the reflective power of the F-P interference spectrum at the Bragg wavelength, current sensing can be realized by using a power meter instead of an expensive OSA for a cheaper solution. Experimental results showed that an optimal sensitivity of 0.8522 nW/A and current measurement resolution of 0.001 A at the Bragg wavelength of 1550 nm with the F-P cavity length of 24 μm was obtained. The proposed current sensing system have advantages of high accuracy and relatively low cost. Furthermore, the usage of FBG can also be helpful to compensate the effect of temperature in further research and applications.
